# FBXW7 promotes autophagy and inhibits proliferation of oral squamous cell carcinoma

**DOI:** 10.1002/iid3.845

**Published:** 2023-05-16

**Authors:** Bo Qiu, Yang Sun, Wei Nie, Qi Yang, Xiangjun Guo

**Affiliations:** ^1^ Dental Clinic Cangzhou Central Hospital Cangzhou Hebei China; ^2^ Dental Department Cangzhou People's Hospital Cangzhou Hebei China

**Keywords:** autophagy, FBXW7, OSCC, proliferation

## Abstract

**Background:**

F‐box and WD repeat domain containing 7 (FBXW7) is a critical tumor suppressor. The expression of FBXW7 is decreased in oral squamous cell carcinoma (OSCC) tissues and shows diagnosis value. We aimed to investigate the influence of FBXW7 overexpression on OSCC cell proliferation and autophagy.

**Methods:**

In Balb/c nude mice, CAL27 xenograft tumor model was established. Western blot was employed to evaluate protein level. Messenger RNA level was analyzed by quantitative reverse transcription‐polymerase chain reaction. Colony formation assay and MTT assay were employed to evaluate cell proliferation.

**Results:**

FBXW7 expression was decreased in OSCC cell lines. FBXW7 inhibited cell proliferation of SCC9 and CAL27. FBXW7 increased Autophagy related 7 (Atg7), Beclin1 (BECN1), B‐cell lymphoma 2 (BCL2) ‐associated X (BAX), BCL2 antagonist killer (BAK), and microtubule‐associated protein 1 light chain 3 (LC3) levels and decreased MCL1 and BCL2 levels in CAL27 cells. FBXW7 decreased tumor volume and weight in CAL27 xenograft tumor model. FBXW7 increased BECN1, Atg7, and LC3 levels in CAL27 xenograft tumor model.

**Conclusion:**

In conclusion, decreased expression of FBXW7 is confirmed in diverse OSCC cell lines. The enhanced FBXW7 expression inhibits cancer cell proliferation and promotes autophagy in both OSCC cells and xenograft tumor model.

## Graphical abstract

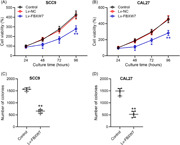

## 1 INTRODUCTION

Oral squamous cell carcinoma (OSCC) is oral and maxillofacial tumor that can be found on the tongue, gums, cheek, floor of the mouth, and palate.^1^, ^2^ Among patients with OSCC, there are more men than women, but the number of female patients has gradually increased in recent years.^3^ Most patients with OSCC are diagnosed in the progressive stage. Despite the development of current treatment technology, the survival rate is still low.^4^ The current treatment for OSCC is mainly based on a combination of surgery and chemotherapy, but it faces the limitation of drug resistance or cross‐resistance in clinical treatment.^5^

Autophagy is degradation process in which damaged organelles and misfolded proteins in the cytosol are involved in the autophagosomes and degraded in lysosomes to keep the normal protein and organelle function.^6^ The correlation between Beclin‐1 (BECN1) monoallelic deletion and enhanced tumor development is proved in both tumor cell lines and mice model.^7^ This is a direct link between tumorigenesis and autophagy. BECN1 participates in the initiation of autophagy and inhibits the migration, invasion, and proliferation of OSCC cell lines.^8^, ^9^ Myeloid cell leukemia‐1 (MCL1) has interaction with BECN1.^10^ The dramatic increase in MCL1 level is triggered by the ablation of BECN1.^7^ Autophagy‐related 7 (Atg7) is also a protein involved in the regulation of autophagy.^11^

F‐box and WD repeat domain containing 7 (FBXW7) acts as a critical tumor suppressor.^12^ It is demonstrated that the expression of FBXW7 influences overall survival and the effect of chemoradiotherapy in OSCC patients.^13^ FBXW7 messenger RNA (mRNA) expression is significantly decreased in OSCC tissues and shows diagnosis value. MCL1 expression is significantly elevated, while BECN1 and Atg7 mRNA expression are significantly lower in OSCC tissues. MCL1 mRNA expression is negatively correlated with FBXW7 mRNA expression, while BECN1 and Atg7 mRNA expressions are positively correlated with FBXW7 mRNA expression. We studied the effects of FBXW7 overexpression on OSCC cell proliferation and autophagy in cell lines and mouse tumor models.

## 2 METHODS

### 2.1 Cells

Human OSCC cell line HSC3, SCC15, SCC25, CAL27, SCC9, and normal oral epithelial cell line CGHNK2 were purchased from ATCC. Cells were cultured in RPMI‐1640 medium with 10% fetal bovine serum (Gibco) and 1% penicillin‐streptomycin (Gibco) with 5% CO_2_ at 37°C.

### 2.2 Transfection

Human FBXW7 complementary DNA reversely‐transcribed from the longest transcript NM_013233 containing all three isoform‐encoding sequences (GAGGATCCCCGGGTACCGGTCGCCACCATGAATC) was cloned into the lentiviral vector GV358 (Genechem) to create the complete functional overexpression plasmid named as Lv‐FBXW7. Another control lentiviral vector was also constructed as Lv‐NC. Conditioned medium containing lentiviruses was harvested 48 h from transfected 293T cells and prepared for further transfection.

### 2.3 Xenograft tumor model

CAL27 xenograft tumor model was set up in Balb/c nude mice. CAL27 cells (5 × 10^6^/mouse) were injected into the flanks of the nude mice. Tumor volumes were calculated at different time points. Tumor volume was calculated using the formula: V = (L × W2)/2 (L is length, W is width). After 4 weeks, mice were killed, and the tumors were carefully removed and weighted. Animal studies were approved by the institutional animal care and use committee of Cangzhou Central Hospital.

### 2.4 Quantitative reverse transcription‐polymerase chain reaction (qRT‐PCR)

Total RNA was isolated by the RNeasy Mini Kit (Qiagen). Reverse transcription was performed by the ReverTra Ace qPCR RT Master Mix (Toyobo). THUNDERBIRD SYBR qPCR Mix (Toyobo) was employed to perform qRT‐PCR. The data were normalized to the internal control, GAPDH to obtain Δ*C*_t_. The final amount of gene of interest relative to control samples was reported by $2^{‐\Delta \Delta C_{t}}$ method. Primers used in this assay were:

FBXW7‐F: ACTGGGCTTGTACCATGTTCA

FBXW7‐R: TGAGGTCCCCAAAAGTTGTTG

Proliferating cell nuclear antigen (PCNA)‐F: GGCTCTAGCCTGACAAATGC

PCNA‐R: GCCTCCAACACCTTCTTGAG

Ki67‐F: AAGCCCTCCAGCTCCTAGTC

Ki67‐R: TCCGAAGCACCACTTCTTCT

GAPDH‐F: TGTTGCCATCAATGACCCCTT

GAPDH‐R: CTCCACGACGTACTCAGCG

### 2.5 Western blot

Western blot was performed with standard protocol. Tumor tissues or cells were homogenized using lysis buffer, followed by determining the concentration using bicinchoninic acid kit. A total of 20 μg protein was separated by SDS–PAGE and transferred to the PVDF membranes. Then, the membranes were blocked using 5% nonfat milk for 90 min at room temperature. The membranes were then incubated overnight at 4°C with primary antibodies. The primary antibodies used in this study were: anti‐FBXW7 (ab109617; Abcam), anti‐BCL2‐associated X (BAX) (14796; CST), anti‐BCL2 antagonist killer (BAK) (6947; CST), anti‐MCL‐1 (5453; CST), anti‐BCL2 (3498; CST), anti‐BECN1 (ab207612; Abcam), anti‐Atg7 (ab133528; Abcam), antimicrotubule‐associated protein 1 light chain 3 (LC3) (ab48394; Abcam), and anti‐GAPDH (5174; CST). Then, the membranes were incubated with HRP secondary antibodies for 1 h at room temperature. The signals were visualized using the chemiluminescence (ECL) kit.

### 2.6 Colony formation assay

In each well of six‐well plate, 1000 cells were seeded. The medium was replaced with fresh medium every 2 days. After 10 days of treatment, the medium was removed and cell colonies were stained with crystal violet (0.1% in 20% methanol).

### 2.7 MTT assay

In each well of 96‐well plate, 5 × 10^3^ cells were cultured. Cell viability was detected using MTT kit (Thermo Fisher Scientific) at 24, 48, 72, and 96 h after the transfection. The culture medium was replaced by MTT for 4 h, avoiding light and at 37°C, and then shaken in dimethyl sulfoxide (DMSO) for 15 min to fully dissolve the formazan crystals. Absorbance of cells was measured at 590 nm with a microplate reader.

### 2.8 Statistical analysis

The data were shown as median (interquartile range). Data were analyzed using the SPSS Version 17.0 (SPSS). Student's *t* test, one‐way analysis of variance (ANOVA), and two‐way ANOVA followed appropriate post hoc test were used to analyze the data.

## 3 RESULTS

### 3.1 The expression of FBXW7 is downregulated in OSCC cell lines

**Figure 1:**
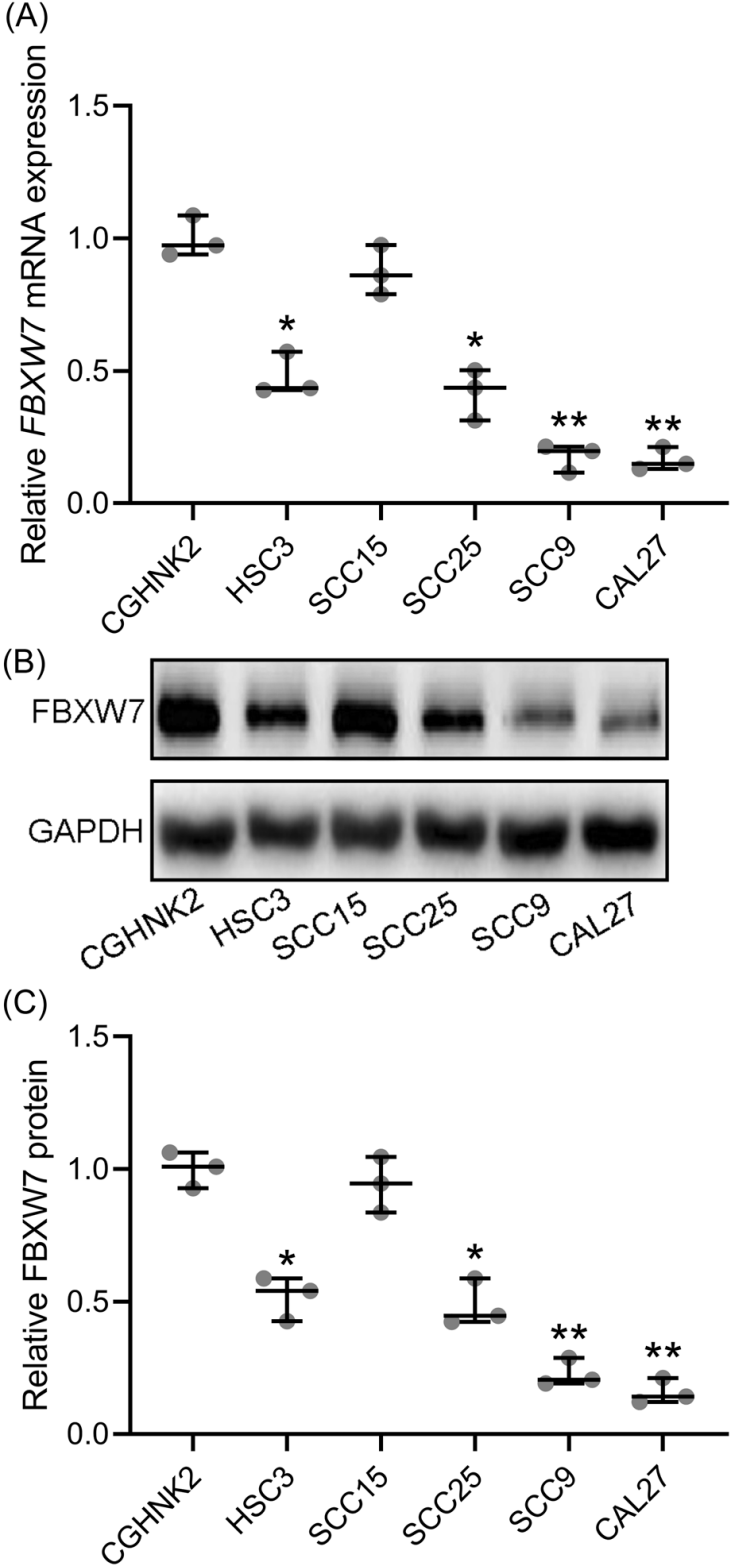
FBXW7 was downregulated in oral squamous cell carcinoma cell lines. qRT‐PCR was used to measure the mRNA levels of FBXW7 among normal oral epithelial cell line CGHNK2 and tongue squamous carcinoma cell line HSC3, SCC15, SCC25, SCC9 and CAL27 (A). Western blot analysis was used to measure the proteins levels of FBXW7 among normal oral epithelial cell line CGHNK2 and tongue squamous carcinoma cell line HSC3, SCC15, SCC25, SCC9, and CAL27 (B). The expressions were normalized to CGHNK2 (C). The data were shown as median (interquartile range). *p < .05; **p < .01 compared with CGHNK2. The experiments were performed in triplicate to confirm the results.

As shown in Figure 1A, FBXW7 mRNA level in HSC3, SCC25, SCC9, and CAL27 cells was dramatically lower than in CGHNK2 cells. Meanwhile, FBXW7 protein level in HSC3, SCC25, SCC9, and CAL27 cells was also significantly lower than in CGHNK2 cells (Figure 1B,C).

### 3.2 FBXW7 inhibits SCC9 and CAL27 cell proliferation

**Figure 2:**
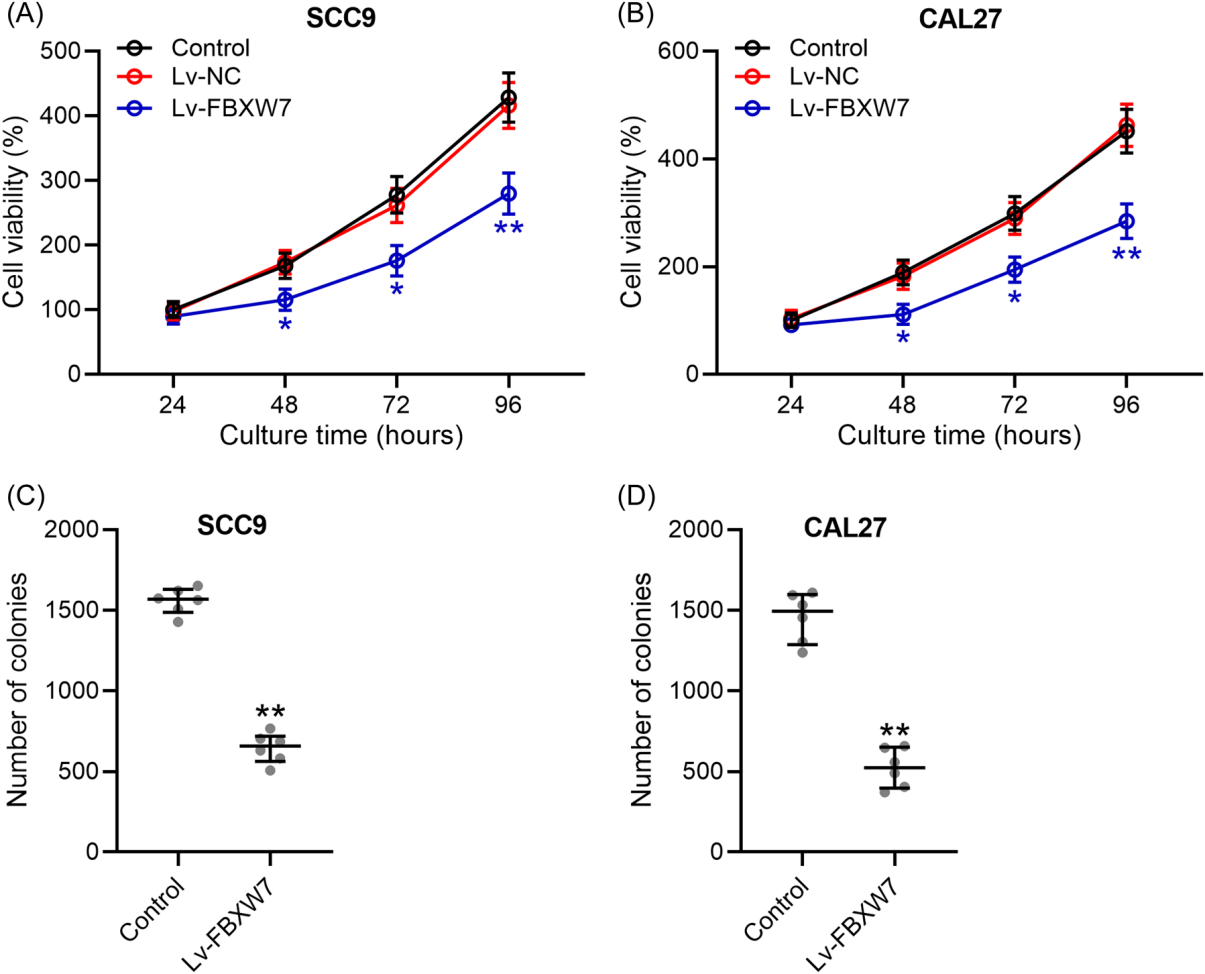
FBXW7 inhibited cell proliferation of SCC9 and CAL27. SCC9 and CAL27 were transfected with LV‐FBXW7 or negative controls. At 24, 48, 72, and 96 h after the transfection, cell viability was measured by MTT (A and B). (C and D) The colony formation assay was conducted 10 days after transfection and the number of colonies were calculated. The data were shown as median (interquartile range). *p < .05; **p < .01 compared with control. The experiments were performed in triplicate to confirm the results.

Based on MTT assay, SCC9 and CAL27 cells transfected with LV‐FBXW7 had significantly inhibited cell viability (Figure 2A,B). A similar finding was observed in colony formation assay (Figure 2C,D). Based on colony formation assay, SCC9 and CAL27 cells transfected with LV‐FBXW7 had significantly decreased number of colonies (Figure 2C,D).

### 3.3 FBXW7 promotes CAL27 cell apoptosis and autophagy

**Figure 3:**
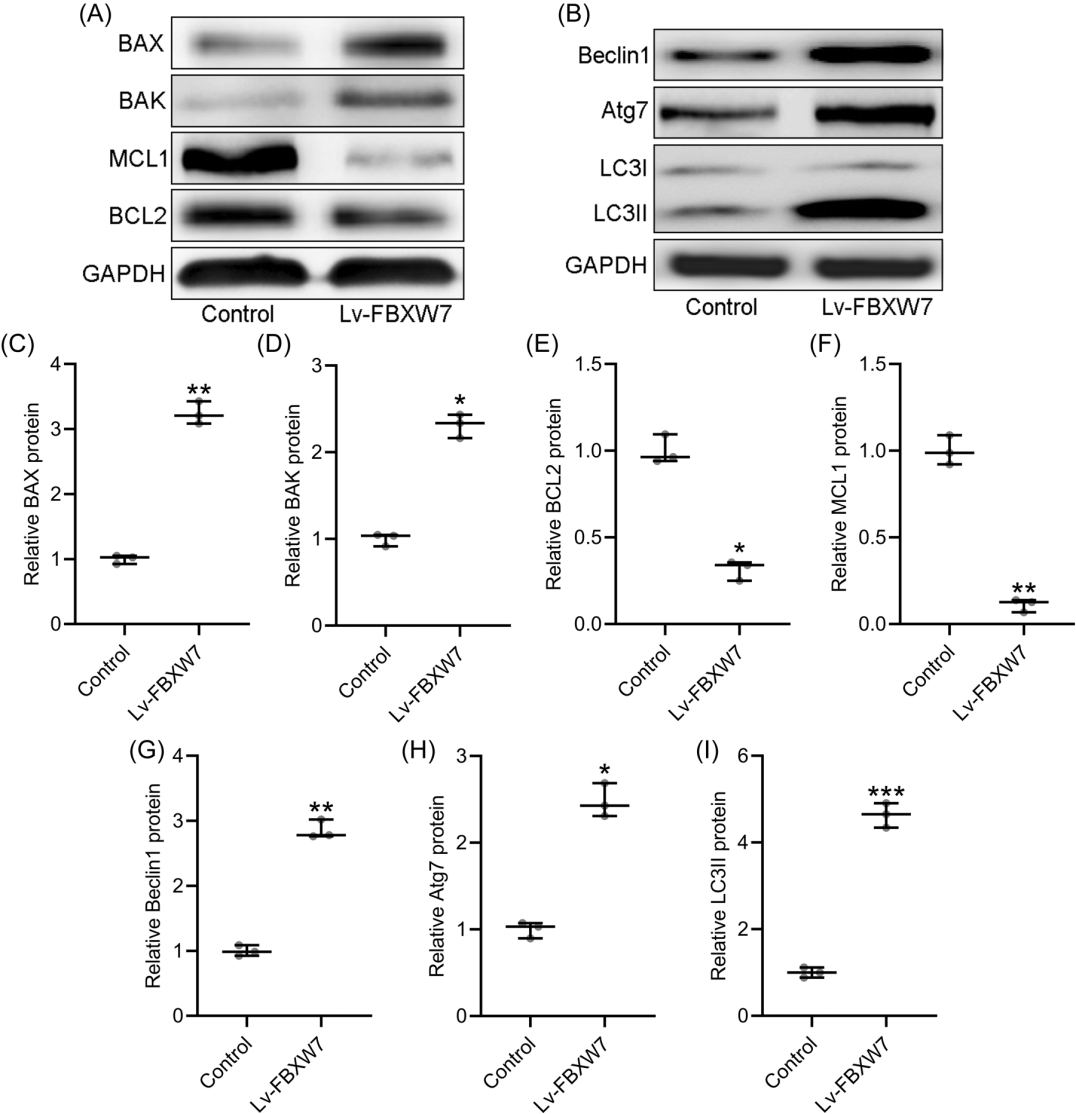
FBXW7 promoted apoptosis and autophagy in CAL27 cells. CAL27 were transfected with LV‐FBXW7 for 72 h. The protein level of proapoptotic molecules BAX, BAK and antiapoptotic molecules MCL1 and BCL2 were examined by western blot (A). The autophagy‐related protein levels of Beclin1, Atg7 and LC3 were examined by western blot (B). The expressions were normalized to control (C–I). The data were shown as median (interquartile range). *p < .05; **p < .01; ***p < .001 compared with control. The experiments were performed in triplicate to confirm the results.

As shown in Figure 3A,C,D, BAX and BAK protein levels were significantly elevated by the overexpression of FBXW7. MCL1 and BCL2 protein levels were significantly declined by FBXW7 overexpression (Figure 3A,E,F). The autophagy‐related protein levels of BECN1, Atg7, and LC3 were also examined by Western blot. FBXW7 overexpression significantly increased BECN1, Atg7, and LC3 protein levels in CAL27 cells (Figure 3B,G–I).

### 3.4 FBXW7 inhibits OSCC cell growth in vivo

**Figure 4:**
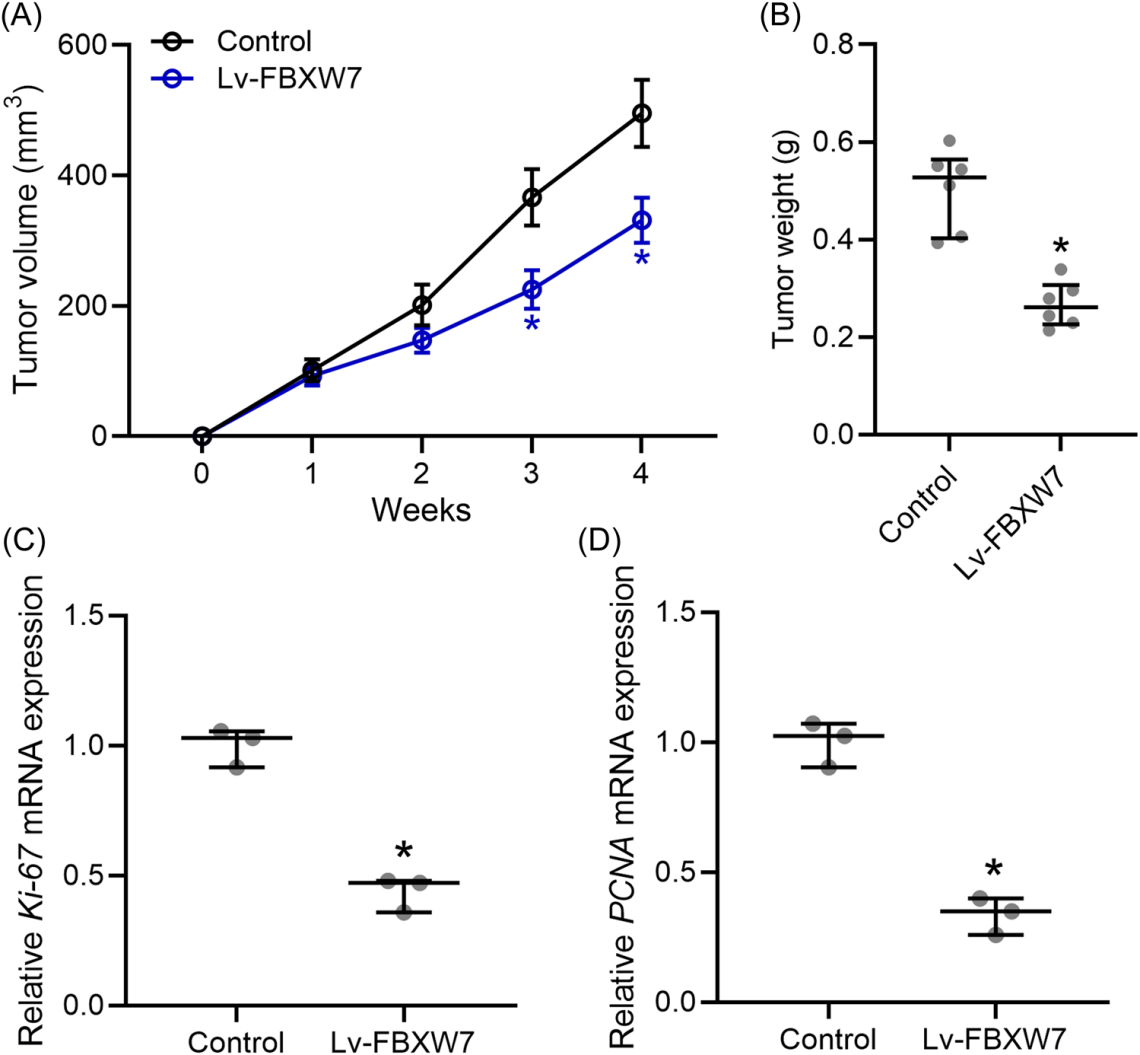
FBXW7 inhibited tongue squamous carcinoma cell growth in vivo. CAL27 xenograft tumor model was set up in Balb/c nude mice. CAL27 cells (5 × 10^6^/mouse) transfected with and without Lv‐FBXW7 were injected subcutaneously into the flanks of the nude mice (*n* = 6 in each group). The tumor growth curve (A) and tumor weight at Day 28 (C) were shown. qRT‐PCR was used to measure the mRNA levels of Ki67 (B) and PCNA (D) in the tumor homogenate from each group (tumor homogenates from six mice in each group were mixed and the experiments were repeated three times). The data were shown as median (interquartile range). *p < .05 compared with control. The experiments were performed in triplicate to confirm the results.

In this research, CAL27 xenograft tumor model was set up in Balb/c nude mice. At 2, 3, and 4 weeks after injection, CAL27 cells transfected with Lv‐FBXW7 had significantly smaller tumor volume than those transfected with Lv‐NC (Figure 4A). At 4 weeks after injection, CAL27 cells with FBXW7 overexpression had significantly lower tumor weight in nude mice (Figure 4B). Ki67 and PCNA mRNA levels in the tumors generated by CAL27 cells transfected with Lv‐FBXW7 were significantly lower than in tumors generated by CAL27 cells transfected with Lv‐NC (Figure 4C,D).

### 3.5 FBXW7 promotes autophagy of tumor tissues in vivo

**Figure 5:**
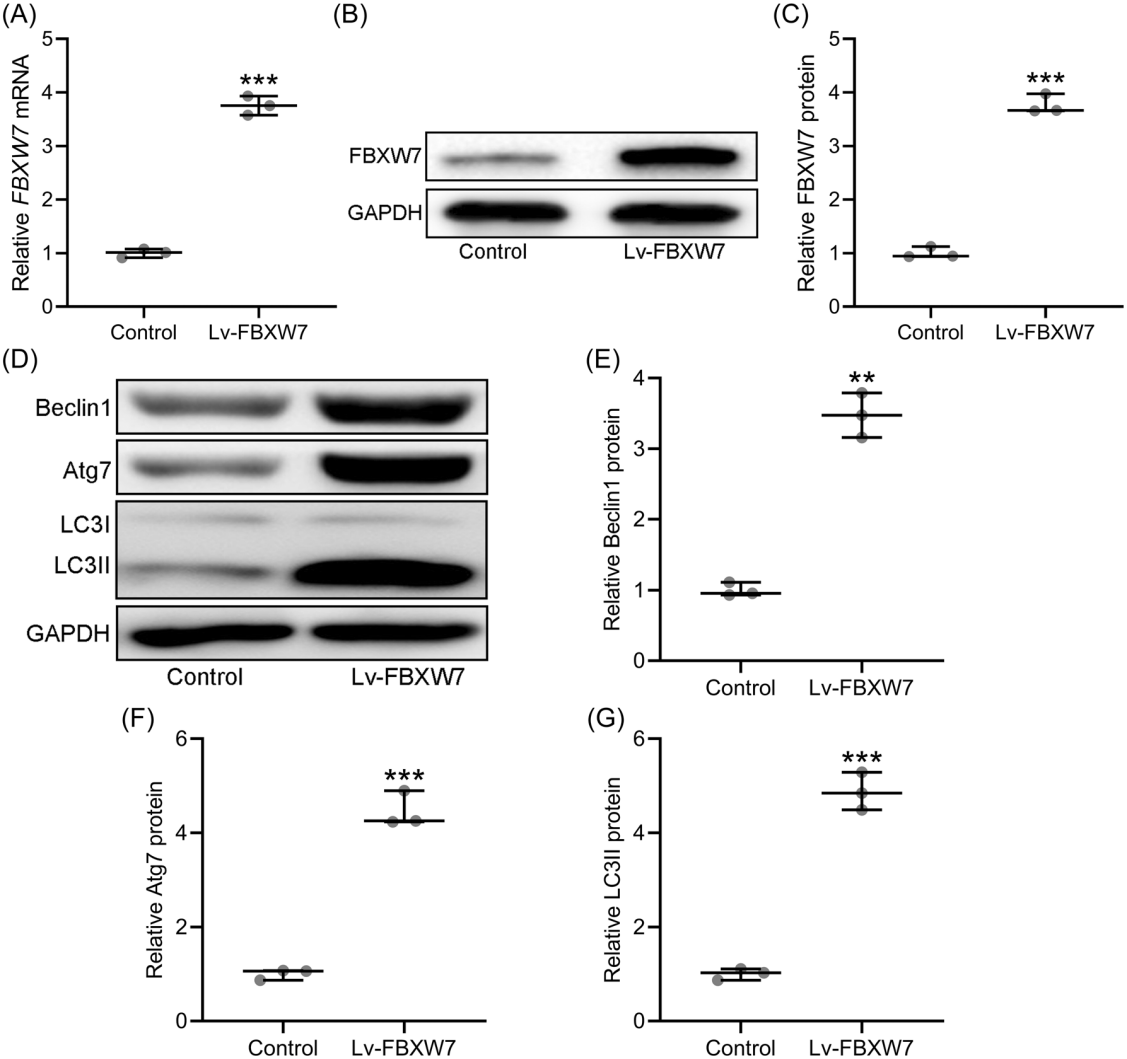
FBXW7 promoted autophagy of tumor tissues in vivo. CAL27 xenograft tumor model was set up in Balb/c nude mice. CAL27 cells (5 × 10^6^/mouse) transfected with and without Lv‐FBXW7 were injected subcutaneously into the flanks of the nude mice (*n* = 6 in each group). After 4 weeks, the tumor homogenate from each group were used for western blot analysis assay and qRT‐PCR. qRT‐PCR was used to measure the mRNA levels of FBXW7 in tumor homogenate from each group (A). The protein levels of FBXW7 between two groups were examined by western blot (B) and the expressions were normalized to control (C). The autophagy‐related protein levels of Beclin1, Atg7, and LC3 were examined by western blot (D). The expressions were normalized to control (E–G) (tumor homogenates from six mice in each group were mixed and the experiments were repeated three times). The data were shown as median (interquartile range). *p < .05; **p < .01; ***p < .001 compared with control. The experiments were performed in triplicate to confirm the results.

In the tumors generated by CAL27 cells transfected with Lv‐FBXW7, FBXW7 expression was significantly higher than in tumors generated by CAL27 cells transfected with Lv‐NC (Figure 5A–C). As shown in Figure 5D–G, tumors with overexpressed FBXW7 had significantly higher protein levels of BECN1, Atg7, and LC3 than tumors in control group.

## 4 DISCUSSION

Autophagy is a cellular stress response to the environment that actively regulates cell survival or death processes during cellular stress, injury, nutritional starvation, aging, and pathogen infection to maintain cells in a stable physiological environment.^14^ Autophagy is regulated by multiple molecules, and proteins encoded by autophagy‐related genes assemble into complexes involved in the whole autophagic process, with unc‐51‐like kinase (ULK) complex responsible for autophagy initiation and vacuolar sorting protein 34 (VPS34) complex responsible for the nucleation of autophagic membranes.^15^, ^16^ Autophagy may inhibit tumor formation by protecting proteins from damage during the tumor formation phase, while in terms of tumor growth, autophagy may promote tumor development by balancing the distribution of metabolic substrates.^17^

Autophagy regulates tumorigenesis through multiple signaling pathways, and the main regulatory pathways of autophagy are phosphatidylinositol‐3 kinase (PI3‐K)/protein kinase B (AKT)/mechanistic target of rapamycin (mTOR), nuclear factor κ B (NF‐κB)/BCL2 and mitogen‐activated protein kinases (MAPK).^18^ AKT in PI3‐Κ‐AΚT‐mTOR signaling pathway can receive signals from PI3‐K and downregulate to mTOR. mTOR inhibits autophagy under normal nutritional conditions, while increases autophagy under starvation.^19^ NF‐κB in the NF‐κB/BCL2 signaling pathway silences apoptotic signaling to keep tumor cells alive. NF‐κB pathway is associated with tumor progression and increased cell migration.^20^ MAPK signaling pathway transmits intra‐ and extracellular signals through enzymatic cascade reactions.

Autophagy has a dual role in oral tumors. The inhibition of autophagy can promote proliferation, invasion and migration of oral cancer cells, thus promote the development of oral cancer. Through chemotherapy, radiotherapy, and other targeted drugs to increase autophagy of oral cancer cells, thereby inhibiting the occurrence and development of oral cancer.

FBXW7, as a key substrate recognition factor in the S‐phase kinase‐associated protein 1 (SKP1)‐cullin 1 (CUL1)‐F‐box protein (SCF) ubiquitin ligase E3 complex, which mediates the ubiquitinated degradation of various proto‐oncogene proteins.^21^ FBXW7 is critical tumor suppressor that is widely involved in various physiological processes including cell proliferation, differentiation, and apoptosis.^12^ The expression of FBXW7 is relatively low in many tumors and FBXW7 mutations are closely associated with tumorigenesis.^21^

Several research have proved the correlation between FBXW7 and the pathogenesis of OSCC.^22^, ^23^, ^24^ In this research, we demonstrated that the expression of FBXW7 was also significantly decreased in OSCC cell line HSC3, SCC25, SCC9 and CAL27. Thus, the pathogenesis of OSCC induces the decreased FBXW7 protein level. In both SCC9 and CAL27 cells, the overexpression of FBXW7 inhibited cell proliferation. Enhanced FBXW7 expression alleviated the viability of OSCC cells.

One of the hallmarks of cancer is the evasion of apoptosis. Apoptosis is regulated by both intrinsic mitochondrial pathway and extrinsic death receptor pathway.^25^ BCL2 family proteins participant in the regulation of intrinsic mitochondrial pathway. In BCL2 family, BCL2 and MCL1 have antiapoptotic effect, while BAX and BAK have proapoptotic effect.^26^ In CAL27 cells, the overexpression of FBXW7 increased the protein levels of BAX and BAK and decreased the protein levels of BCL2 and MCL1. Thus, FBXW7 promoted the apoptosis of OSCC cell line.

LC3 participants in the biogenesis of autophagosomes through the conjugation of phosphatidylethanolamine (PE).^27^ This lipidation process is performed by Atg7, Atg3, and other proteins and converts LC3 form soluble form to membrane‐bound form.^28^ As a coiled‐coil protein, BECN1 interacts with BCL2 and is involved in BECN1‐PIK3C3‐PIK3R4 complex which plays a crucial function in autophagy induction.^29^ In CAL27 cells, the overexpression of FBXW7 increased the protein levels of BECN1, Atg7, and LC3. Thus, FBXW7 promoted the autophagy of OSCC cell line.

In this research, CAL27 xenograft tumor model was set up in Balb/c nude mice. Tumors generated by CAL27 cells with enhanced FBXW7 expression had smaller volume and weight than those generated by control CAL27 cells. Ki67 and PCNA are markers of proliferation in the diagnosis of OSCC.^30^ The decreased Ki67 and PCNA mRNA levels in tumors with FBXW7 overexpression further confirmed the effect of FBXW7 in the inhibition of OSCC development in mice model. The overexpression of FBXW7 also increased the protein levels of BECN1, Atg7, and LC3 in tumors generated by CAL27 cells. FBXW7 promoted the autophagy in CAL27 xenograft tumor model.

This research also has some limitations. We have proved that FBXW7 regulate tumor cell autophagy through the regulation of the protein levels of BECN1, Atg7, and LC3. The specific molecular mechanism of FBXW7 in the regulation of relative protein levels is still not be investigated. In this research, the function of FBXW7 was proved in tumor cell lines and xenograft tumor model. In future work, the function of FBXW7 should be further explored in animal models and patients.

In conclusion, decreased expression of FBXW7 is confirmed in diverse OSCC cell lines. The enhanced FBXW7 expression inhibits cancer cell proliferation and promotes autophagy in both OSCC cells and xenograft tumor model.

## AUTHOR CONTRIBUTIONS

**Bo Qiu**: Conceptualization (lead); data curation (lead); writing—original draft (lead); writing—review and editing (lead). **Yang Sun**: Data curation (supporting); writing—original draft (supporting); writing—review and editing (supporting). **Wei Nie**: Data curation (supporting); writing—original draft (supporting); writing—review and editing (supporting). **Qi Yang**: Data curation (supporting); writing—original draft (supporting); writing—review and editing (supporting). **Xiangjun Guo**: Data curation (supporting); writing—original draft (supporting); writing—review and editing (supporting).

## CONFLICT OF INTEREST STATEMENT

The authors declare no conflict of interest.

## ETHICS STATEMENT

Animal studies were approved by the institutional animal care and use committee of Cangzhou Central Hospital.

## Data Availability

Data could be obtained upon reasonable request to the corresponding author.
